# Ultrasound assessment of gastric content in patients undergoing laparoscopic cholecystectomy after preoperative oral carbohydrates: a prospective, randomized controlled, double-blind study

**DOI:** 10.3389/fsurg.2023.1265293

**Published:** 2023-09-04

**Authors:** Yali Ge, Dejuan Shen, Yinyin Ding, Keting Wu, Yang Zhang

**Affiliations:** ^1^ Department of Anesthesiology, Yangzhou University Affiliated Northern Jiangsu People’s Hospital, Yangzhou, China; ^2^ Department of Ultrasound, Northern Jiangsu People’s Hospital, Yangzhou, China

**Keywords:** carbohydrates, cholecystectomy, laparoscopic, gastric emptying, gastroparesis

## Abstract

**Background:**

To evaluate the gastric volume and nature after drinking preoperative oral carbohydrates in patients undergoing laparoscopic cholecystectomy via ultrasonography.

**Methods:**

One hundred patients who had been scheduled for elective laparoscopic cholecystectomy were enrolled and randomized into the traditional fasting group (Control group, *n* = 50) and the carbohydrate group (CHO group, *n* = 50). Patients in the Control group fasted solids and drink from midnight, the day before surgery. Patients in the CHO group drank 800 ml and 400 ml of oral carbohydrates 11 and 3 h before surgery, respectively. At 2 h after oral carbohydrates (T_1_), all patients underwent an ultrasound examination of residual gastric contents; if the patients had a full stomach, the assessment was performed again 1 h later (T_2_). A stomach containing solid contents or >1.5 ml/kg of liquid was considered “full”. The primary outcome was full stomach incidences at the above time points. The secondary outcomes included gastric antral CSA in the right lateral decubitus (RLD) and semi-sitting positions, as well as gastric volume (GV), GV per weight (GV/kg), and Perla's grade at T_1_.

**Results:**

Compared with the Control group, the incidence of entire stomach was significantly high in the CHO group 2 h after oral carbohydrates. At the T_1_ time point, 6 patients (13.3%) in the Control group and 14 patients (30.4%) in the CHO group presented with a full stomach [95% confidence interval (CI), (0.96–5.41), *P* = 0.049]. At T_2_, 3 patients (6.7%) in the Control group and 4 patients (8.7%) in the CHO group had a full stomach, with no marked differences between the two groups [95% CI, (0.31–5.50), *P *= 0.716]. Compared with the Control group, CSA in the semi-sitting and RLD positions, GV and GV/W were significantly high in the CHO group at T_1_ (*P < *0.05). The median (interquartile range) of the Perlas grade was 1 (0–1) in the Control group and 1(1–1.25) in the CHO group (*P* = 0.004).

**Conclusion:**

Cholecystectomy patients experience a 2 h delay in gastric emptying after receiving preoperative carbohydrates. In LC patients, the fasting window for oral carbohydrates before surgery should be adequately prolonged.

**Clinical Trail registration:**

Chinese Clinical Trail Registry, No: ChiCTR2200055245.

## Introduction

Cholelithiasis is a common digestive system disease with incidences of about 10% with an annual increase of 0.60%–1.39% (1). Currently, laparoscopic cholecystectomy (LC) is the main treatment option for gallstone disease (2). Despite following the strict fasting guidelines, about 13% of patients scheduled for LC still have a full stomach (3). This is higher than the reported incidences of a full stomach in the general surgical population (2.7%–6.2%) (4, 5), which may be attributed to decreased gastrointestinal peristalsis caused by gallstones and inflammation of surrounding tissues in cholelithiasis patients (1).

In 1999, Professor Dr. Henrik Kehlet proposed Enhanced Recovery After Surgery (ERAS) (6), it refers to the application of the evidence of Evidence-based medicine, optimization of perioperative treatment, cooperation among clinical disciplines to achieve the best therapeutic effect, and in this process, to minimize the pain of patients and trauma caused by surgery, reduce the incidence of postoperative complications, and shorten the length of hospital stay. Since then, multimodal programs that are based on the ERAS concept, which is widely practiced in general surgeries (e.g., gastrointestinal surgery) and other surgical fields, have been developed to overcome the perioperative morbidity challenges (6, 7). Preoperative oral carbohydrate ingestion is an important component of ERAS protocols. Experimental and clinical studies have highlighted the significant advantages of preoperative oral carbohydrates, such as decreasing postoperative insulin resistance, improving glucose metabolism, promoting intestinal functions, and enhancing postoperative recovery in patients undergoing various elective surgeries (6–9). Recent randomized and controlled studies have focused more on the safety of preoperative oral carbohydrates. These studies support that administration of preoperative oral carbohydrates up to 2 h before general anesthesia induction or surgery does not increase the incidence of perioperative complications, including regurgitation and aspiration in most elective surgeries (9). Moreover, since carbohydrate drinks consist of polymers with low osmolality, gastric emptying will not be delayed after carbohydrate drinking, and it can be administered up to 2 h before anesthesia or surgery, apart from patients with obvious gastric emptying disorders (8–10). As we previously indicated, cholelithiasis patients may experience decreased gastrointestinal peristalsis, although there is no published data on gastric emptying following preoperative oral carbohydrate loading in patients undergoing LC.

Gastric ultrasonography, a non-invasive, simple approach that can be performed at the patient's bedside with little discomfort, does not expose the patients to radiation. Gastric ultrasonography provides reliable quantitative and qualitative data regarding stomach contents prior to anesthesia (11). Gastric ultrasound can accurately reflect gastric content volume by measuring the cross-sectional antral area (12). Therefore, it is a practical imaging technique for evaluating gastric contents qualitatively and quantitatively throughout the perioperative period.

We used gastric ultrasonography to evaluate the gastric volume and nature after drinking preoperative oral carbohydrates in patients undergoing laparoscopic cholecystectomy and assess whether the fasting period following carbohydrate intake could be extended to enhance patient safety and reduce the risk of a full stomach before anesthesia induction. To achieve this, we compared the correlations among gastric ultrasound measurements, including cross-sectional area (CSA), gastric volume (GV), the likelihood of a full stomach, and other measurements, between patients who fasted and patients who ingested carbohydrate-containing fluids before surgery.

## Methods

### Study design

This prospective, double-blind, randomized, placebo-controlled trial was approved by the institutional ethical committee of Northern Jiangsu People's Hospital (No. 2021ky285, Chairperson: JJ Qian) on 18 November 2021, and it was performed from March 2022 to July 2022. A written informed consent was obtained from all participants. Before patient enrolment, this trial was registered at ClinicalTrials.gov (ChiCTR2200055245).

Cholelithiasis patients that were scheduled for elective laparoscopic cholecystectomy surgery, who were aged between 18 and 64 years old and in physical status I or II according to the American Society of Anesthesiologists (ASA) were enrolled in this study. The exclusion criteria included pregnancy, a co-occurring illness that prevents gastric emptying (such as obesity, diabetes, hiatal hernia, gastroesophageal reflux disease, ileus, or enteral tube feeding), a prior gastrointestinal surgery history, psychiatric or mental disorders, alcoholism, or drug abuse.

#### Perioperative management

The day before surgery, patients in both groups abstained from ingesting solid foods after 20:00. Patients in the Control group abstained from drinking after 21:30 while patients in the CHO group were given 800 ml of carbohydrate-containing fluid [SuQian, NO-NPO, (12.5% Maltodextrin; 52 kcal/100 ml, 260 mOsm/kg; Jiangsu, China)] at 21:30 and 400 ml of carbohydrate-containing fluid from 5:00 to 5:30. All selected patients were the first ones in the operation room and received anesthesia induction at 8:30. At 1 h before anesthetic induction, intramuscular injections of 0.005 mg/kg glycopyrrolate were performed.

#### Study protocol

**Figure 1:**
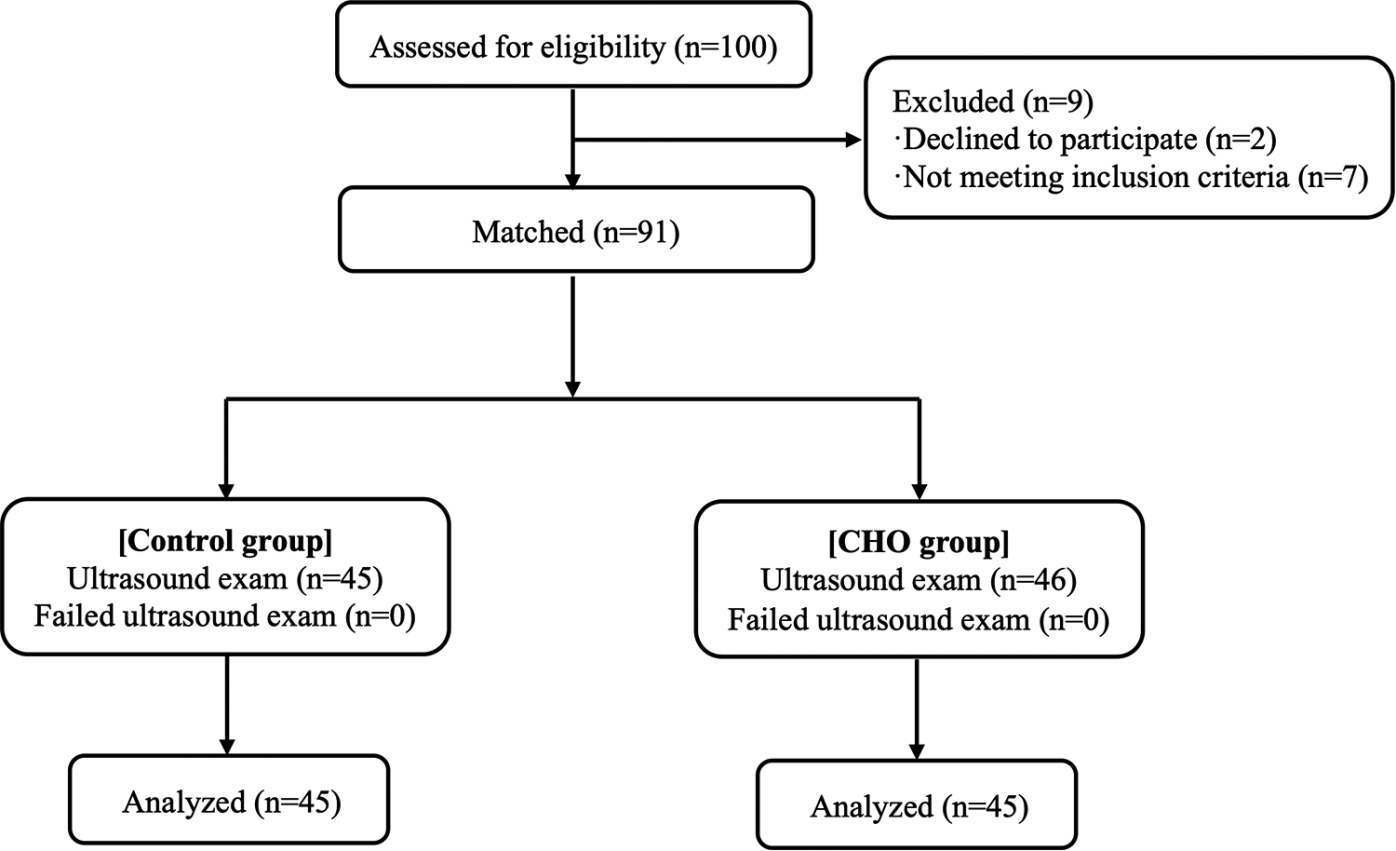
Study protocol.

Moreover, before anesthesia induction, all patients were subjected to gastric ultrasound examination using an ultrasound system with a 2–5 MHz curved array transducer (Sonosite Ultrasonic Fujifilm Investment Co., LTD, China). Ultrasonographic assessments were performed when patients were in supine position, semi-sitting position (45° head up), and right lateral decubitus (RLD) positions, respectively (13). The left lobe of the liver, the pancreas, the inferior vena cava, and the superior mesenteric vein were used as markers for locating the gastric antrum in the epigastric region of the parasagittal plane. The gastric antrum was situated just behind the left lobe of the liver and in front of the pancreas, while the transducer was positioned along the sagittal plane of the epigastric region. The pancreas was located behind the inferior vena cava. To ascertain the presence of any gastric contents and what kind of contents were present in the gastric sinus, the images were first subjected to a qualitative examination. When the gastric antrum was flat and collapsed, it was defined as empty (Figure 1A) and when the antrum was a distended cavity, it was deemed to have some clear fluid or solid contents. Generally, ground glass appearance with low echo was seen as transparent liquid (Figure 1B), while appearance with medium echo was regarded as solid contents (Figure 1C). After establishing the absence of solid contents, patients were categorized using the Perlas grading scale (14) (grade 0: the antrum appears empty in both the supine and RLD positions; grade 1: clear fluid is only appreciable in the RLD position; grade 3: clear fluid is appreciable in both the supine and RLD positions). The Perlas grading scale does not apply to patients whose antrum has solid contents.

For quantitative assessments, the probe was tilted clockwise or counterclockwise to obtain the most minor, round-shaped, cross-sectional view of the antrum. The cross-sectional areas (CSA) of the antrum in both semi-sitting position and RLD position were calculated using the formula of the area of an ellipse: CSA = (AP × CC  × *π*)/4 (AP = anteroposterior diameter and CC = cranio-caudal diameter) (15). The diameters were measured from serosa to serosa between contractions. Gastric volume (GV) was calculated using the formula as reported by Perlas et al. (16): GV (RLDP) = 27.0 + 14.6 × CSA (at RLDP)−1.28 × age. A stomach was defined as “empty” if the gastric antrum was classified as Perlas grade 0 or gastric contents were liquid with GV ≤ 1.5 ml/kg. If the stomach contained solid contents or >1.5 ml/kg of liquid, it was considered “full” (17).

First, ultrasound examinations in the CHO group were conducted 2 h after carbohydrate ingestion (T_1_ and at the corresponding time points in the control group). If patients were evaluated as having a full stomach at T_1_, they were assessed again by ultrasound 1 h later (T_2_). The primary outcome was the incidence of a full stomach at the above time points. The secondary outcomes included gastric antral CSA in right lateral decubitus (RLD) and semi-sitting positions, as well as gastric volume (GV), GV per weight (GV/kg), and Perla's grade at T_1_.

If patients still had a full stomach at T_2_, they were subjected to a standardized anesthesia induction protocol to prevent aspiration by reflux to ensure patient safety. Preoxygenation was performed for 3 min, avoiding manual positive pressure ventilation. Anesthetic drugs were sequentially and rapidly administered (intravenous midazolam 0.08–0.10 mg/kg, rocuronium bromide 0.9–1.2 mg/kg, sufentanil 0.3–0.5 µg/kg and isoproterenol 1.5–2.0 mg/kg), and when the patients lost consciousness and spontaneous respiration, cricoid cartilage pressure was applied. Finally, tracheal intubation was performed (18).

After gastrointestinal ultrasonography assessment, hunger, thirst, and satisfaction levels were measured using the visual analog scale, ranging from 0 (not at all) to 10 (very much), in the waiting area. Patients' preoperative adverse reactions, such as dizziness, panic, and anxiety were also recorded. The frequency of postoperative nausea and vomiting at 24 h, hospital stay time, and incidences of perioperative reflux aspiration as well as postoperative rapid rehabilitation surgery-related variables were noted.

#### Statistical analysis

The SPSS software version 26 (IBM Inc., Armonk, NY, USA) was used for the statistical analyses. Data are expressed as mean ± standard deviation, medians (interquartile range), or as numbers (%). The standardized mean difference was used to compare baseline data between the randomized groups (SMD). A point estimate of CSA in the semi-sitting and RLD positions, as well as a 95% confidence range, were reported (CI). The 2-sample *t*-test and the Wilcoxon rank-sum test were used to assess the treatment effects on normally distributed continuous variables and Perlas grade. Randomized groups were compared on the incidence of patients with full a stomach using a *χ*^2^ test. A value of *P* < 0.05 was considered statistically significant.

#### Sample size estimation

During the pre-test study, we collected gastric volume data from the control and CHO groups. Full stomach incidences at 2 h after oral carbohydrates were 12% and 36%, respectively. Using the PASS 11.0 software, the two groups were set up at a 1:1 ratio, setting *α* = 0.05 and 1-*β* = 0.8; a sample size of 48 cases per group was calculated. To reduce on the possible sample loss and increase the statistical accuracy, a final decision was made to include 100 patients in this study.

## Results

### Patient characteristics

**Figure 2:**
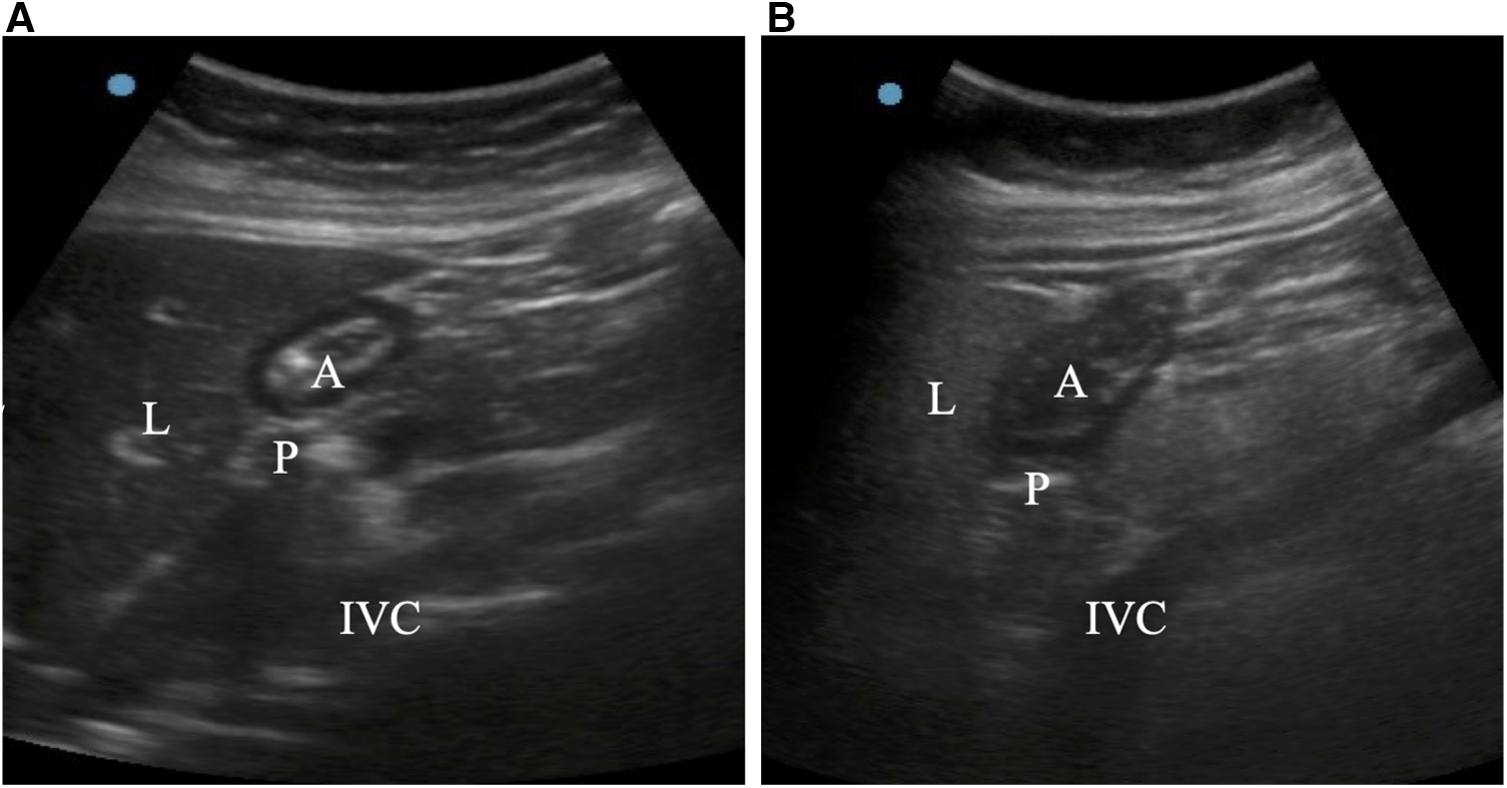
Ultrasound images of the stomach antrum when it was in the right lateral decubitus position. In an empty stomach, the gastric antrum appears flat and folded (A). If fluid content is present, the stomach antrum appears swollen and hypoechoic (B). A = antrum; IVC = inferior vena cava; L = liver; P = pancreas.

Between October 2021 and February 2022, 100 patients were enrolled. Among them, 7 participants were excluded because they did not get the designated interventions while 2 participants were excluded because their data were incomplete. Therefore, 91 patients were included and randomized into the control or CHO groups. All participants adhered to the fasting guidelines and did not exhibit any negative side effects (Figure 2). Table 1 shows the baseline characteristics of the study population. The CHO group's fasting period for solid meals was 12.50 h, whereas the Control group's was 13.00 h. The CHO group fasted for 2 h while the Control group fasted for 10 h (Table 1).

**Table 1:** Patient characteristics.

	Control group (*N *= 45)	CHO group (*N *= 46)
Age (y)	48 (12)	46 (10)
Sex (M/F)	17/28	23/23
Height (cm)	165.3 (8.9)	166 (7.8)
Weight (kg)	60 (55–74.5)	65 (60–75)
BMI (kg/m^2^)	23.4 (2.7)	24.2 (2.5)
ASA physical status (I/II)	37/8	38/7
Fasting time for clear liquids (h)	10.00 (6.75–11.00)	2.00 (1.50–2.75)
Fasting time for solids (h)	13.00 (11.50–14.00)	12.50 (11.00–13.50)
Diagnosis		
Calculous cholecystitis	37 (82.2)	36 (78.3)
Acalculous cholecystitis	4 (8.9)	5 (10.9)
Cholangitis	4 (8.9)	5 (10.9)
Type of surgery		
LC	41 (91.1)	42 (91.3)
LC + LCBDE	4 (8.9)	4 (8.7)

Data are presented as mean (standard deviation), number of patients (%), or medians [interquartile range].

ASA, American society of anesthesiologists; BMI, body mass index; LC: laparoscopic cholecystectomy; LCBDE: laparoscopic common bile duct exploration.

### Gastric volume

In supine, semi-sitting, and RLD positions, the stomach antrum could be seen in all patients. At T_1_ (2 h after oral carbohydrate), 32 patients (69.6%) in the CHO group showed an empty stomach: 6 patients presented with grade 0 antrum, 24 patients presented with grade 1 antrum, and 2 patients presented with grade 2 antrum. Fourteen patients (30.4%) had a full stomach: 4 patients had solid contents and 2 patients had grade 1 antrum. Eight patients with grade 2 antrum had gastric fluid volume of >1.5 ml/kg. At T_1_, 39 patients (86.7%) in the Control group had an empty stomach: 16 patients presented with grade 0 antrum and 23 patients presented with grade 1 antrum. Six patients (13.3%) had a full stomach: 3 patients had solid contents and 3 patients with grade 2 antrum had a gastric fluid volume of >1.5 ml/kg [95% confidence interval (CI), (0.96–5.41), *P* = 0.049]. Compared with the Control group, CSA in the semi-sitting and RLD positions, GV and GV/W were significantly high in the CHO group at T_1_ (*P < *0.05). The median (interquartile range) of the Perlas grade was 1 (0–1) in the Control group and 1 (1–1.25) in the CHO group (*P* = 0.004). At T_2_ (3 h after oral carbohydrates), 4 patients (8.7%) in the CHO group and 3 patients (6.7%) in the Control group had a full stomach, with no significant differences in full stomach incidences between the two groups [95% confidence interval (CI), (0.31–5.50), *P *= 0.716; Table 2].

**Table 2:** Comparison of gastric volume.

	Control group (*N *= 45)	CHO group (*N *= 46)	Difference (95% CI)	*P*-value
Quantitative analysis at T_1_				
CSA (RLD, cm^2^)	6.2 (1.9)	7.1 (2.0)	−0.93 (−1.77, −0.09)	0.031^a^
CSA (supine, cm^2^)	3.9 (0.84)	4.5 (1.1)	−0.60 (−1.02, −0.18)	0.006^a^
Gastric volume (ml)	55.5 (33.2)	70.9 (31.3)	−15.4 (−29.4, −1.4)	0.031^a^
Gastric volume (ml/kg)	0.8 (0.5)	1.1 (0.4)	−0.20 (−0.10, −0.4)	0.043^a^
Qualitative assessment at T_1_				0.004^c^
Grade0	16/45 (35.6)	6/46 (13.0)		
Grade1	23/45 (51.1)	26/46 (56.5)		
Grade2	3/45 (6.7)	10/46 (21.7)		
Solids	3/45 (6.7)	4/46 (8.7)		0.716^b^
Patients with a full stomach(T_1_,T_2_)				
T_1_	6 (13.3)	14 (30.4)	–	0.049^b^
T_2_	3 (6.7)	4 (8.7)	–	0.716^b^

Values are presented as mean (standard deviation) or number of patients (%), Differences are (fasting - carbohydrate).

CSA, cross-sectional area; CI, confidence interval; RLD, right lateral decubitus; SD, standard deviation, T_1_= 2 h after oral administration of carbohydrates, T_2_= 3 h after oral administration of carbohydrates, There were 3 patients in the control group and 4 patients in the CHO group with solid gastric ultrasound imaging, which were not included in the quantitative assessment of gastric ultrasound at T_1_.

^a^ Student's t test.

^b^ *χ*^2^ test.

^c^ Wilcoxon rank-sum test.

### Preoperative hunger, thirst, satisfaction scores in visual analog scale and postoperative adverse reactions and postoperative rapid recovery-related indicators

The degrees of preoperative hunger, thirst, and satisfaction are presented in Table 3. Compared with the Control group, the CHO group exhibited a decrease in preoperative hunger and thirst scores and improved satisfaction scores (*P *< 0.05). During anesthesia induction, no patient in either group suffered from regurgitant aspiration. Postoperative nausea and vomiting incidences were lower, and time to first exhaust was earlier in the CHO group, compared with the Control group (*P <* 0.01). Differences in operative time between the groups were not significant (Table 3).

**Table 3:** Preoperative hunger, thirst, satisfaction scores in visual analog scale and postoperative adverse reactions and postoperative rapid recovery-related indicators.

	Control group (*N *= 45)	CHO group (*N *= 46)	*P*-value
Hunger score	5.5 ± 2.3	4.0 ± 1.7	0.034
Thirst score	5.8 ± 2.1	2.8 ± 1.1	<0.001
Satisfaction score	2.9 ± 1.7	6.5 ± 1.5	<0.001
Nausea, vomiting	11 (24.4)	4 (8.7)	0.043
Reflux aspiration	0 (0)	0 (0)	1.000
Time to first exhaust (min)	723.57 (77.54)	550.76 (65.43)	<0.001
Operative time (d)	2.3 (0.4)	2.2 (0.5)	0.693

Data are presented as mean (standard deviation) or number of patients (%).

## Discussion

The ERAS protocol and related guidelines recommend that patients without contraindications receive 400 ml of oral carbohydrate ingestion within 2 h prior to surgery. Compared with the general surgical population, cholelithiasis patients are accompanied by prolonged gastric and gallbladder emptying, as evidenced by high incidences of a full stomach after fasting (3, 19). The ideal pre-operative fasting period following carbohydrate consumption in cholelithiasis patients has not yet to be determined. In our pilot trial, patients were subjected to a gastric ultrasound 2 h after oral carbohydrates. To assess gastric emptying of these cholelithiasis patients, patients with a full stomach underwent sinus ultrasound evaluation again 1 h later, which was up to 3 h after oral carbohydrate intake. This trial revealed that: First, cholelithiasis patients exhibited a delayed gastric emptying time; Second, when fasting time after POC treatment was extended to 3 h, there was no greater danger of full stomach in cholelithiasis patients; Third, patients undergoing LC may benefit from preoperative oral carbohydrates by having less postoperative nausea, vomiting and quickly recovering.

We used gastric ultrasound to measure the anteroposterior and cephalocaudal diameters of gastric sinus when patients were in semi-sitting and RLD positions to calculate CSA, which were introduced in the Perlas model to respectively calculate GV. In the semi-sitting position, GV had a small range of applicability and was used as a reference indicator, whereas it had a wide range of applicability in the RLD position and was highly accurate, even in mildly obese people. Therefore, in our study, the GV model in the RLD position was used as the primary index for assessing gastric emptying of preoperative oral carbohydrates in cholelithiasis patients. In our investigation, the mean projected stomach volume for grade 2 was 1.7 ml/kg, consistent with other results (1.7 ml/kg for pregnant women (20), 1.6 ml/kg for very obese patients (21), and 1.8 ml/kg for patients undergoing general surgery (4)). Therefore, using 1.5 ml/kg as the threshold for assessing a full stomach in our study is reliable.

Patients in the CHO group were accompanied by much higher ultrasound indices of the gastric sinus region, including CSA, GV, and GV/W, as well as a higher Perlas Grade at 2 h after carbohydrate ingestion. Regarding antral grades distribution in this study, proportions of grade 0 (35.6%), grade 1 (51.1%), and grade 2 (6.7%) in the Control group were comparable to those of the general population in previous studies (2, 22). However, proportions of Perlas Grade 1 (56.5%) and Grade 2 (21.7%) in the CHO group were significantly higher than those of the Control group. Sinus ultrasound revealed that there were 14 patients with a full stomach in the CHO group after 2 h carbohydrate ingestion and 4 patients in the Control group at the corresponding time point. Full stomach incidence in the CHO group was about 30%, which was higher than the 13% in the Control group, proving that slowing down of gastric peristalsis in cholelithiasis patients may trigger a decrease in gastric emptying functions to some extent. The higher incidence of full stomach in the CHO group after 2 h carbohydrate ingestion might be related to cholelithiasis-associated pathophysiological changes, including hormone-free stimulation of diseased gallbladder mucosa to mediate gastric motility, reflex pyloroduodenal reflux due to inflammation in and around the gallbladder, or delayed gastric emptying due to inflammatory adhesions around the gallbladder (23). Ciaula et al. (1) assessed 46 patients with gallstones, 24 patients after LC, and 65 healthy volunteers as the control group. The assessments included dyspepsia and ultrasonic examination of gastric emptying since disease onset. Compared with healthy volunteers, patients with gallstones had significantly higher scores for dyspepsia, gastric emptying, and prolonged intestinal transport. Gallstones can lead to motor dysfunctions of the whole intestines (including the esophagus, stomach, small intestines, and colon), resulting in prolonged gastric emptying (23). In this study, patients with a full stomach and who had been evaluated by gastric ultrasound 2 h after oral carbohydrate ingestion were examined again 1 h later. It was found that 3 h after oral carbohydrate drinks, there were 3 and 4 patients with full stomachs in the CHO and Control groups, respectively. Differences in final incidences of full stomach between the two groups were insignificant (8.7% vs. 6.7%). Patients with final full stomach before anesthesia induction in the two groups received rapid sequence induction with 30° head up tilt, and none of the patients had reflux aspiration. There was a significant risk of a full stomach after 2 h of oral carbohydrate intake in cholelithiasis patients. However, when the abstinence window was extended to 3 h, the risk for preoperative full stomach for patients with cholelithiasis was significantly reduced, which may have some positive implications in improving the security of patients with cholelithiasis drinking carbohydrates preoperatively.

Due to advances in ERAS, preoperative oral carbohydrates (POC) are widely used to improve the quality of postoperative recovery. The main components of preoperative carbohydrates used in this study are maltodextrin and polydextrose. Maltodextrin is an extremely absorbable complex polysaccharide that can effectively reduce digestive stress in patients with gastric emptying disorders and promote postoperative gastrointestinal function recovery (24). Polydextrose is a soluble fiber that can effectively increase surimi volume in the gastrointestinal tract, increase the patient's sense of satiety, slow down carbohydrates absorption and enhance the patient's subjective satisfaction. Therefore, POC treatment may be beneficial for reducing hunger and thirst, and increasing subjective satisfaction, as well as reducing postoperative nausea and vomiting incidence. Meanwhile, the time of first postoperative gastric emission for patients in the CHO group was much earlier than in the control group, consistent with previous studies (25).

The ERAS protocol recommends 400 ml of oral carbohydrates 2 h before surgery. Our results preliminarily suggested that preoperatively taking 400 ml carbohydrates orally 2 h before anesthesia induction can lead to a higher incidence of full stomach in patients undergoing cholecystectomy. To ensure regular gastric emptying of LC patients, another similar study suggested that contents of oral enzymatic ice powder solution should be reduced to 300 ml (less than the routine 400 ml) when given to LC patients 2 h preoperatively (26). Therefore, it is recommended that during preoperative preparation of patients undergoing LC, potential gastric emptying disorders should be considered. For safety concerns, some procedural adjustments should be taken when patients undergoing cholecystectomy preoperatively receive oral carbohydrates. Based on our results and other studies, we postulated that fasting time after carbohydrate drinks ingestion in LC patients should be appropriately extended (e.g., up to 3 h), or the volume of carbohydrate solution be reduced (e.g., to 300 ml). For patients with high-risk factors for a full stomach before anesthesia induction, the stomach capacity should be assessed by ultrasound (27). If patients with full stomach determined by ultrasound examination, the operation time should be appropriately delayed in selective surgery, or some protective schemes, such as appropriate airway control devices, rapid sequential induction, and placement of gastric tube suction should be put in place to prevent regurgitation (24, 25).

There are some limitations in this study. First, this was a single-center prospective controlled study, lacking multi-center clinical evidence. Multi-center studies should be performed to further verify our findings. Second, after drinking carbohydrates, we did not measure the gastric capacity at different time points, therefore, the gastric emptying time of carbohydrates could not be determined. Finally, since this study was conducted in the Asian community, our findings may not be generalizable to other ethnic communities. The physiological differences among different ethnic groups may have a certain impact on the results of this study, this is due to potential racial variations in the pathophysiology of gallbladder illnesses.

## Conclusions

The LC patients may experience extended gastric emptying. We suggest that to improve the safety of POC treatment in patients undergoing LC, the fasting time window for oral carbohydrates can be appropriately extended in patients who receive POC treatment before surgery (e.g., up to 3 h). If necessary, bedside ultrasound should be used before inducing anesthesia to evaluate the gastric contents.

## Data Availability

The raw data supporting the conclusions of this article will be made available by the authors, without undue reservation.
